# Comparison of the therapeutic effects of endoscopic submucosal dissection and minimally invasive esophagectomy for T1 stage esophageal carcinoma

**DOI:** 10.1111/1759-7714.13203

**Published:** 2019-09-25

**Authors:** Lei Gong, Jie Yue, Xiaofeng Duan, Hongjing Jiang, Hongdian Zhang, Xi Zhang, Zhentao Yu

**Affiliations:** ^1^ Department of Esophageal Cancer, Tianjin's Clinical Research Center for Cancer and Key Laboratory of Cancer Prevention and Therapy, National Clinical Research Center for Cancer Tianjin Medical University Cancer Institute and Hospital Tianjin China; ^2^ Department of Periodontal Dentistry, School and Hospital of Stomatology Tianjin Medical University Tianjin China

**Keywords:** Endoscopic submucosal dissection, esophageal cancer, minimally invasive esophagectomy, squamous cell carcinoma

## Abstract

**Background:**

In recent years, diagnosis of early squamous cell carcinoma of the esophagus has been increasingly emphasized. The application of endoscopic submucosal dissection (ESD) has enabled safe resection of esophageal lesions. Minimally invasive esophagectomy (MIE) is also safe and feasible for early stages of the cancer. This study aimed to compare the therapeutic effects of early esophageal carcinoma treatment, and find the best predictive factor for the selection of treatment for T1a patients.

**Methods:**

We performed a retrospective study of early‐stage patients admitted to Tianjin Medical University Cancer Institute and Hospital between January 2015 and December 2018. A total of 128 patients underwent MIE, while 78 patients underwent ESD. The depth of the tumor invasion, lymph node metastasis, and complications were compared between the two groups.

**Results:**

In the ESD group, 76.92% of the patients were stage T1a, while 34.38% in the MIE group were stage T1a. The lymph node metastasis rate was 16.41% in the MIE group (6.98% in T1a stage), which related to tumor differentiation, tumor length (≥37.5 mm), depth of invasion, and angiolymphatic invasion. However, the R0 resection rate was only 73.08% in the ESD group. Comprehensive analysis of all T1 patients in the two groups revealed that the positive margin was related to tumor differentiation, tumor width (≥13.5 mm), and depth of invasion (≥3.25 mm).

**Conclusion:**

For early‐stage cases, lymph node metastasis and positive margins are risk factors affecting long‐term survival. Efficient predictive factors mentioned in our study would provide a proper indication for treatment strategy selection.

## Key points

### Significant findings of the study

Our study compared the surgical effects of ESD and MIE. We found tumor differentiation, tumor length, depth of invasion, and angiolymphatic invasion can be used as predictors for lymph node metastasis.

### What this study adds

In our study, the R0 resection rate was low. We conducted a comprehensive study of two groups. We found out that tumor differentiation, tumor width, and depth of invasion were predictive factors for positive margins.

## Introduction

Esophageal cancer (EC) is one of the most common types of cancer types, ranking seventh in incidence and sixth in mortality worldwide.^1^ Esophagectomy is still the standard treatment for esophageal cancer. In recent years, the application of minimally invasive esophagectomy (MIE) has shown the potential for advantages over open surgery.^2^ According to the results of the TIME trial in 2012, less pulmonary infection, less blood loss, and a better quality of life were found in the MIE group than in the open surgery group.^3^ However, severe postoperative complications were still not ineluctable.

With the recent progress in endoscopic techniques, the incidence of superficial esophageal squamous carcinoma (ESCC) is increasing.^4^ For treatment of early‐stage ESCC, endoscopic submucosal dissection (ESD) has been shown to be the better treatment option. ESD could offer en bloc resection with a lower local recurrence rate compared with endoscopic mucosa resection (EMR).^5^ It is also a less invasive treatment strategy with a shorter hospitalization period.^6^

On the other hand, with regard to the anatomy of lymphatic drainage, the inner layers (mucosa and submucosa) and the outer layers (muscularis propria and adventitia) of the thoracic esophagus are quite different, which results in lymph node metastasis (LNM) in the very early stage.^7^ It has been reported that the rate of LNM from M1‐SM1 was 0.0–18.2%.^4^ Mucosal infiltration can be an important indication for ESD, while subinfiltration can be treated surgically due to the increased risk of lymph node metastasis (LNM).^6^

In addition, en bloc R0 resection was correlated with local recurrence for esophageal cancer patients who received ESD.^5^ Endoscopic ultrasonography (EUS) is an accurate method for determining the depth of tumor invasion.^8^ The use of EUS is feasible for the classification of T category ESCC. However, it should be used with caution for the classification of stages Tis, T1a, and T1b.^9^

Overall, the aim of this study was to compare MIE and ESD with regard to the management of stage T1 ESCC. We retrospectively analyzed the pathological risk factors and results from EUS. Previous studies only analyzed the data of EUS and pathological findings within the ESD group, where most cases were at T1a stage with negative vertical margins. In our study, we also attempted to include the data from T1b patients in the MIE group and provide a comprehensive evaluation of the mucosal status of early tumors. These factors could form the basis of the future guidelines for the selection of treatment for superficial ESCC.

## Methods

### Patient selection

We retrospectively analyzed patients who underwent ESD or MIE for ESCC at Tianjin Medical University between January 2015 and December 2018. Patients with stage T0, T1a, and T1b ESCC were included in the study. Esophageal cancer was confirmed by endoscopic biopsy before the treatment. The patients underwent a computed tomography (CT) scan of the chest and epigastrium with contrast. This study was approved by the Ethics Committee of Tianjin Medical University Cancer Institute and Hospital.

### ESD and MIE

The ESD procedure was performed in hospitalized patients under general anesthesia with trachea intubation in the left lateral decubitus position. The lesion was dyed with Lugol solution and marked with an electrosurgical generator. Fluid with 0.4% sodium hyaluronate acid was injected into the submucosal layer to mark the border. The submucosa was then dissected en bloc by an experienced endoscopist. The specimen was then spread smoothly and fixed by pins on a corkboard, soaked in formalin, and sent for pathological examination.^10^ The muscular layer was then carefully checked for any additional injury and a nasogastric tube was placed.

All MIE patients underwent McKeown resection with a two‐field lymphadenectomy. MIE included thoracoscopy, thoracoscopy combined with laparoscopy, or robot‐assisted thoracoscopy. The entire stomach was used for the reconstruction. A gastric tube 3 to 5 cm wide was used for the McKeown operation and transhiatal esophagectomy with cervical anastomosis. In this study, five cases underwent MIE after ESD due to pathologically positive ESD margin.

### Pathological assessment

Pathological assessment was performed by the Department of Pathology. Tumor description included tumor location, tumor size, depth of invasion, and differentiation. The depth of invasion was divided into M1 (intraepithelium layer), M2 (lamina propria layer), M3 (muscularis mucosa layer), and SM1‐3 (submucosal layer).^11^ R0 resection was defined as without SM invasion, positive margin resection, and lymphovascular invasion. Tumor differentiation was classified into three groups: well, moderate, and poor. Pathological results were evaluated according to the eighth edition of TNM staging.

### Statistical analysis

Statistical methods used in this study include the Student's *t*‐test (or Mann‐Whitney U test) and Fisher's exact test (or Pearson's chi‐square test). Univariate logistic regression and multivariate logistic regression analysis were used to identify risk factors and independent predictors of LNM and submucosal invasion, respectively. Continuous data are presented as mean ± SD. To determine the performance of the predictive models, receiver operating characteristics (ROC) curves were constructed, and the areas under the curve (AUCs) and 95% confidence intervals (CIs) were calculated. All *P*‐values were two‐sided, and the significance level was set at *P* < 0.05. All analyses were performed in SPSS (SPSS 19.0 for Windows, SPSS Inc., Chicago, IL, USA).

## Results

### Patient characteristics

A total of 206 patients were included in our study. Of these, 78 and 128 underwent ESD and MIE, respectively. The baseline characteristics of patients who underwent either ESD or MIE are shown in Table 1. The histological types of the two groups were all squamous cell carcinoma. There were no significant differences in age, gender, tumor location, or Charlson comorbidity index between the two groups. However, the depth of tumor invasion was significantly different with the ESD group having more M1 lesions, while the MIE group had more SM1‐3 lesions (*P* = 0.000).

**Table 1:** Baseline patient characteristics

	ESD (78)	MIE (128)	*P*‐value
Age (years)	60.54 ± 8.70	59.50 ± 7.06	0.350
Gender			0.852
Male	65(83.33%)	105(82.03%)	
Female	13(16.67%)	23(17.97%)	
Tumor location			0.174
Upper thoracic	7(8.97%)	4(3.13%)	
Middle thoracic	40(51.28%)	74(57.81%)	
Lower thoracic	31(39.75%)	49(38.28%)	
Charlson Comorbidity Index			0.277
0	10(12.82%)	8(6.25%)	
1	16(20.51%)	33(25.78%)	
2	38(48.72%)	56(43.75%)	
3	10(12.82%)	17(13.28%)	
≥4	4(5.13%)	14(10.94%)	
Depth of tumor invasion			
M1	49(62.82%)	16(12.50%)	0.000*
M2	2(2.56%)	4(3.13%)	
M3	9(11.54%)	24(18.75%)	
SM1‐3	18(23.08%)	84(65.62%)	

* Statistically significant.

ESD, endoscopic submucosal dissection; MIE, minimally invasive esophagectomy.

### Pathological features

Most of the lesions in the ESD group were restricted to the mucosa layers (76.92%) (Table 2). However, there were still some stage T1b cases found in the endoscopic group. According to the pathological results, there were two cases with the appearance of positive LNM (2.56%) in the submucosal layer. Meanwhile, the MIE seemed to be the correct procedure for the T1b patients (65.62%) when ESCC is first diagnosed. However, the total LNM rate for the N1‐2 category increased to 16.41%, which indicated the necessity of lymphadenectomy for patients at this stage. When we compared the pT category with tumor differentiations, the tumor invasion showed a positive correlation with the degrees of differentiation. Furthermore, the TNM staging was significantly different between the two groups (*P* = 0.045). However, 29.4% of patients who exceeded stage 1A were in the ESD group. This may be a potential risk factor for future recurrence and metastasis of ESCC.

**Table 2:** Pathological outcomes

	ESD (78)	MIE (128)	*P*‐value
pT category			0.000*
pT1a	60(76.92%)	44(34.38%)	
pT1b	18(23.08%)	84(65.62%)	
pN category			0.017*
pN0	76(97.44%)	107(83.59%)	
pN1	2(2.56%)	20(15.63%)	
pN2	0(0)	1(0.78%)	
Lymph node numbers	NA	23.53	
Differentiation			0.000*
Well	59(75.64%)	40(31.25%)	
Moderate	17(21.79%)	71(55.47%)	
Poor	2(2.56%)	17(13.28%)	
pTNM			0.045*
IA	55(70.51%)	26(20.31%)	
IB	21(26.92%)	81(63.28%)	
IIB	2(2.56%)	20(15.63%)	
3A	0(0)	1(0.78%)	

* Statistically significant.

### Postoperative outcomes

The overall postoperative complication rates were 23.08% in the ESD group and 24.22% in the MIE group (Table 3), with no significant difference (*P* = 1.000) being found between the groups. The return ICU rates increased from 2.56% to 7.03% when we compared the ESD group with the MIE group (*P* = 0.213). The postoperative pneumonia rate also increased from 2.56% to 5.47% according to different treatment protocols (*P* = 0.488). The rate of esophageal perforation was 5.13% in the ESD group, and the rate of anastomotic leakage was 3.91% in the MIE group (*P* = 0.732). There were three cases of stenosis in the endoscopic groups, while there was only one case in the surgical resection group. Nine (8.97%) patients in the ESD group suffered from a pneumothorax. Recurrent laryngeal nerve injury was only found in the MIE group (7.03%).

**Table 3:** Operative data and postoperative outcomes

	ESD	MIE	*P*‐value
R0 resection (%)	73.08%(57)	100%(128)	0.000*
Hospital stay (days)	10.17 ± 7.91	24.15 ± 9.41	0.000*
Cost ($)	3673.77 ± 1002.83	22 272.30 ± 25 630.59	0.000*
Complication	18(23.08%)	31(24.22%)	1.000
Return ICU	2(2.56%)	9(7.03%)	0.213
Pneumonia	2(2.56%)	7(5.47%)	0.488
Perforation/leakage	4(5.13%)	5(3.91%)	0.732
Stenosis	3(3.85%)	1(0.78%)	0.333
Pneumothorax	7(8.97%)	0(0)	0.333
RLN injury	0(0)	9(7.03%)	0.333

* Statistically significant.

Perforation/leakage, perforation for ESD group, leakage for MIE group.

RLN, recurrent laryngeal nerve.

ESD is a less invasive surgical treatment, which resulted in a much shorter hospital stay compared with patients who underwent MIE (*P* = 0.000) (Table 3). The overall expenses for the treatment of T1 stage esophageal cancer patients were also significantly different. The average cost in the ESD group was 3673.77 ± 1002.83 dollars compared with 22 272.30 ± 25 630.59 dollars in the MIE treatment (Table 3). As previously mentioned in the ESD group, 23.08% of the patients were stage T1b and the R0 resection rate was only 73.08%. Some patients underwent MIE after ESD.

### Relationship between the pathological results and LNM

**Figure 1:**
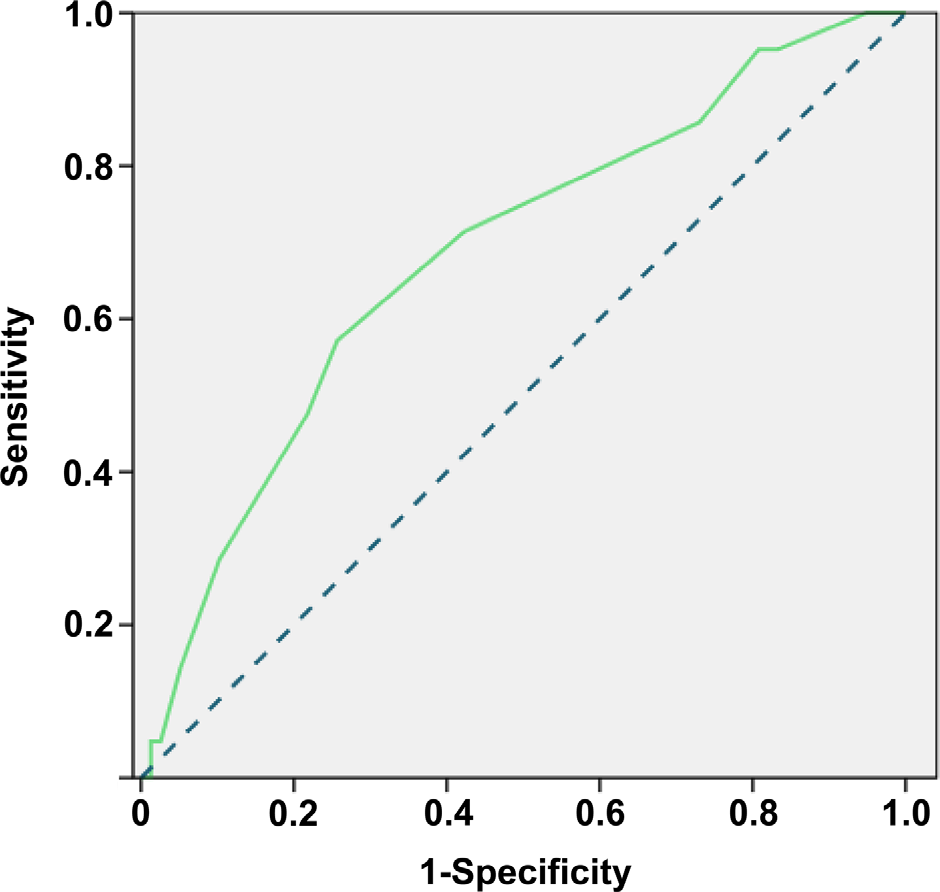
Performance of lymph node metastasis predictive models using ROC curves. (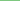) Tumor length.

In our study, the LNM rate was approximately 16.42% (Table 2). Univariate analysis indicated that larger tumor length resulted in the prevalence of LNM (Table 4) (negative = 29.67 ± 22.94 vs. positive = 43.33 ± 24.31, *P* = 0.019). Furthermore, poor tumor differentiation (well vs. moderate and poor, *P* = 0.020), deeper tumor invasion (M1‐3 vs. SM1‐3, *P* = 0.027), and angiolymphatic invasion (*P* = 0.035) caused tumor‐positive lymph nodes. Figure 1 showed the predictive values of the tumor length on the ROC curves. According to the cutoff point, we determined that a tumor length of 37.5 mm was one of the factors in the multivariate analysis. The remaining parameters were tumor differentiation, tumor invasion, and angiolymphatic invasion. Only tumor length (*P* = 0.005) and tumor invasion (*P* = 0.048) were independent predictors for LNM. Other characteristics, including age (*P* = 0.843) and tumor location (*P*= 0.534), were not associated with LNM (Table 5).

**Table 4:** Univariate analysis of the risk factors for lymph node metastases

	LNM −	LNM +	*P*‐value
Age	59.56 ± 7.19	59.23 ± 6.52	0.843
Tumor location			0.534
Upper	3	1	
Middle/lower	103	21	
Differentiation			0.020*
Well	37	2	
Moderate and poor	70	19	
Tumor length (mm)	29.67 ± 22.94	43.33 ± 24.31	0.019*
Depth of invasion			0.027*
M1‐3	35	3	
SM1‐3	65	19	
Angiolymphatic invasion	Length		0.035*
Absent	104	19	
Present	2	3	

* Statistically significant.

LNM, lymph node metastases.

**Table 5:** Multivariate analysis of the risk factors for lymph node metastases

	OR	(95% CI)	*P*‐value
Differentiation	0.626	0.142–2.764	0.536
Tumor size	0.121	0.041–0.359	0.000*
Depth of invasion	0.194	0.042–0.887	0.034*
Angiolymphatic invasion	0.137	0.019–1.004	0.050

* Statistically significant.

CI, confidence interval; OR, odds ratio.

### Pathological features based on invasion depth

**Figure 2:**
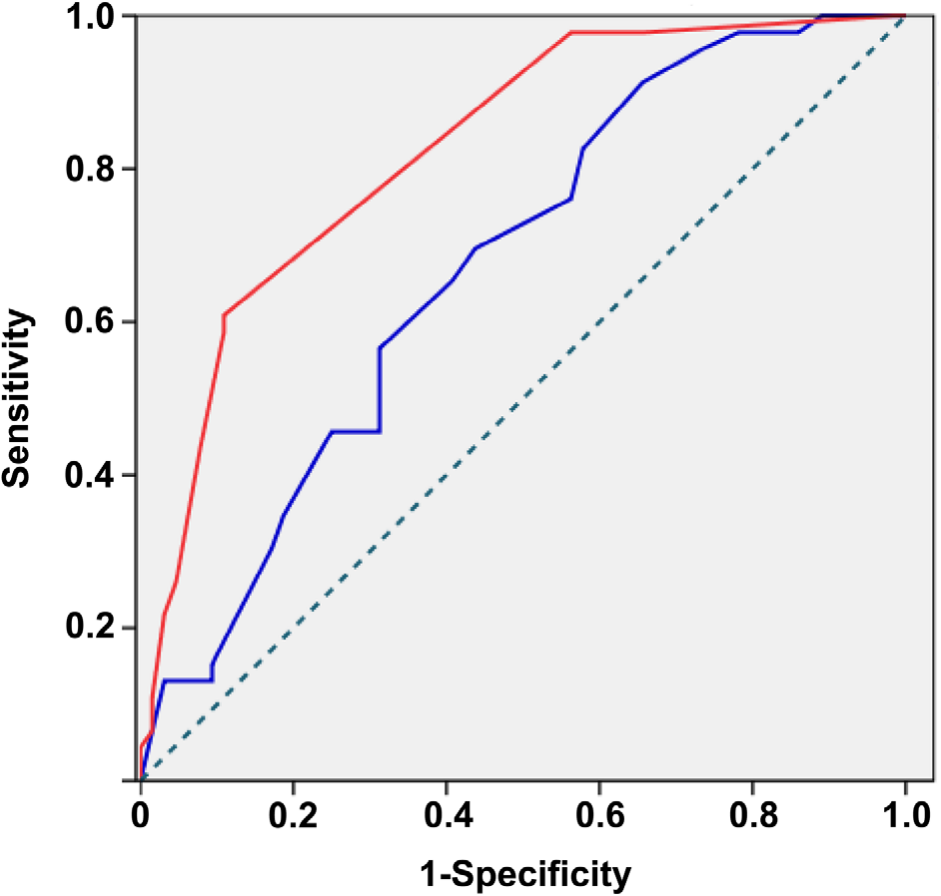
Performance of submucosal invasion predictive models using ROC curves. (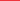) Tumor width and (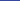) depth of invasion

The R0 resection rate was low in the ESD group (73.08%). In our study, we summarized patients from both groups who received EUS before treatment; the total number of patients was 128. In this study, we added the data of EUS and pathologic findings from stage T1b patients with positive vertical margins as we thought it likely that this could increase the sensitivity and specificity of the predictive factors in comparison with existing studies. Univariate analysis showed that the tumor width (*P* = 0.004) and tumor thickness (*P* = 0.000) under the evaluation of EUS, along with tumor differentiation (*P* = 0.000) were important indicators when patients received endoscopic resection. ROC curves of the tumor width and tumor thickness were used again to calculate the critical value for patients, and those who were suitable underwent ESD treatment. According to the cutoff points, tumor width of 13.5 mm, tumor thickness of 3.5 mm, and with well‐defined tumor differentiation are the best indicators for T1a patients suitable for ESD treatment (Fig 2; Table 6).

**Table 6:** Univariate analysis of the risk factors for submucosal invasion

	SMI− (*n* = 106)	SMI + (*n* = 22)	*P*‐value
Age	60.37 ± 7.306	60.60 ± 7.30	0.885
Tumor location			1.000
Upper	4	3	
Middle/lower	63	42	
Differentiation			0.000*
Well	57	5	
Moderate and poor	10	40	
Tumor length (mm)	30.52 ± 24.02	33.42 ± 30.16	0.602
Tumor width (mm)	16.81 ± 12.24	24.98 ± 16.99	0.004*
Depth of invasion (mm)	3.11 ± 1.84	4.71 ± 2.26	0.000*

* Statistically significant.

SMI, submucosal invasion.

## Discussion

ESCC is the major pathological type in East Asian patients with a poor prognosis. With the improvement of diagnostic techniques, more and more patients are being diagnosed at an early stage. Either ESD or MIE can be used as a treatment for T1 stage patients. However, ESD is only suitable for stage T1a disease, while MIE is a good choice for stage T1b disease due to LNM. Furthermore, EUS is an efficient way to determine the T category.^9^ According to previous studies, clinicopathologic factors such as tumor location and length and depth of invasion affected preoperative evaluations and the efficiency of the treatment.^4^, ^5^, ^12^

Compared with MIE, ESD was a minimally invasive operation. Endoscopic resection for the mucosal lesion could perfectly reserve the normal anatomical structure of the digestive system.^13^ Patients would never suffer from postoperative reflux or weight loss. This would largely preserve patient short‐ and long‐term quality of life. Although the application of MIE largely reduced postoperative complications,^14^ severe complication rates in the patients who underwent ESD were even lower.^15^ In addition, hospital stay duration and the total cost were much less in the ESD group compared with the MIE group. These factors might be important when considering patients with comorbidities or those advanced in age.

However, ESD was only a local treatment. According to previous studies, there was a low risk of LNM (0–7.7%), even in esophageal cancer limited to the mucosa. When the tumor invaded into the submucosa, the LNM rate varied from 36.4% to 50%.^16^, ^17^, ^18^, ^19^ In our study, the metastasis rate was 6.98% for stage T1a and 22.09% for stage T1b. Based on these findings, endoscopic resection is a less invasive treatment for T1a patients, and MIE with radical lymphadenectomy would benefit T1b patients at greater risk of LNM.

As previously mentioned, patients underwent comprehensive evaluations before treatment. However, it was hard to make an accurate diagnosis of the disease. For stage T1, EUS is the best noninvasive tool with a sensitivity of 85% and specificity of 87%.^8^ However, for the evaluation of LNM, we could not find an efficient method. In our study, we found that tumor differentiation, tumor length, depth of tumor invasion, and angiolymphatic invasion were correlated with the presence of positive lymph nodes. Tumor length and depth of tumor invasion were independent risk factors. Tumor length exceeding 37.5 mm with moderate or poor tumor differentiation are considered predictive factors for LNM. These findings were quite similar to the findings of previous studies,^4^, ^13^ However, tumor length more than 20 mm was considered the independent predictive factor for LNM in the study by Zhou *et al*.^4^

From the published literature, complete resection rates of ESD varied from 78%–100%^20^, ^21^; the R0 resection rate was only 73.08% in our study. The major reason for this was positive vertical margins. Whether it is suitable for patients with M3‐SM1 tumor invasion to undergo EDS is still controversial. To further identify this question, we combined the clinical data of the two groups and performed a comprehensive analysis of the pathological results and preoperative EUS evaluations. As reported above, tumor width, tumor thickness, and tumor differentiation were related to submucosa invasion. A similar report from Hazama *et al*. showed that tumors in the left esophageal wall and tumors measuring >1/2 of the esophageal circumference were predictors of difficult esophageal ESD.^5^ In adenocarcinoma, a superficial infiltration less than 20 mm is thought to be an oncologically adequate alternative to surgery.^22^ However, in our study, we also included the data from T1b patients as we were of the opinion this could offer enough positive vertical margin cases, and establish a comprehensive evaluation from T1a to T1b disease. The predictive factors which we achieved could be more accurate.

There are several limitations to our study. First, we did not include the overall survival rate of the two groups due to the time limit; a further description will be provided in future studies. Second, we only performed a retrospective analysis in a single tertiary cancer center. A prospective clinical trial based on the parameters detailed in this study may be carried out in the future. Third, the study sample size was not large enough, but the data we obtained was sufficient to draw significant conclusions.

In conclusion, efficient predictive factors for the evaluation of LNM and tumor invasion are very important for determining the treatment strategy. A comprehensive analysis of tumor length, tumor width, tumor thickness, and tumor differentiation is essential before patients receive any kind of surgical treatment. Using the predictive factors from our study could largely increase the R0 rates and decrease the LNM rates for patients who undergo ESD. Proper selections for the treatment of T1 esophageal cancer could benefit quality of life and the long‐term survival of patients.

## Disclosure

No authors report any conflict of interest.
